# Fuzzy optimization for identifying anti-cancer targets with few side effects in constraint-based models of head and neck cancer

**DOI:** 10.1098/rsos.220633

**Published:** 2022-10-26

**Authors:** Feng-Sheng Wang, Pei-Rong Chen, Ting-Yu Chen, Hao-Xiang Zhang

**Affiliations:** Department of Chemical Engineering, National Chung Cheng University, Chiayi, Taiwan

**Keywords:** flux balance analysis, genome-scale metabolic model, cancer cell metabolism, drug discovery, hybrid differential evolution, multi-level optimization

## Abstract

Computer-aided methods can be used to screen potential candidate targets and to reduce the time and cost of drug development. In most of these methods, synthetic lethality is used as a therapeutic criterion to identify drug targets. However, these methods do not consider the side effects during the identification stage. This study developed a fuzzy multi-objective optimization for identifying anti-cancer targets that not only evaluated cancer cell mortality, but also minimized side effects due to treatment. We identified potential anti-cancer enzymes and antimetabolites for the treatment of head and neck cancer (HNC). The identified one- and two-target enzymes were primarily involved in six major pathways, namely, purine and pyrimidine metabolism and the pentose phosphate pathway. Most of the identified targets can be regulated by approved drugs; thus, these drugs are potential candidates for drug repurposing as a treatment for HNC. Furthermore, we identified antimetabolites involved in pathways similar to those identified using a gene-centric approach. Moreover, *HMGCR* knockdown could not block the growth of HNC cells. However, the two-target combinations of (*UMPS*, *HMGCR*) and (*CAD*, *HMGCR*) could achieve cell mortality and improve metabolic deviation grades over 22% without reducing the cell viability grade.

## 1. Introduction

Head and neck cancers (HNC) are among the most common types of cancer worldwide [1,2]. HNC is characterized by a high locoregional recurrence rate and includes cancers of the oral cavity, oropharynx, hypopharynx and larynx. People who smoke, abuse alcohol or chew betel nuts are at greater risk of developing HNC than people who do not have these habits [3]. The incidence of oral cancer (excluding lip cancer) is high in South and Southeast Asia, Western and Eastern Europe, Latin America, and the Caribbean and Pacific regions. Taiwan (part of East Asia) has one of the world's highest incidence rates of oral cancers [3]. Most HNC cases are squamous cell carcinoma. Recent advances in molecular biology have enabled the documentation of substantial genetic differences between head and neck squamous cell carcinoma (HNSCC) cells and normal cells, potentially enabling the development of new therapeutics and chemoprevention methods [4].

The efficient identification of drug targets and their side effects is a key challenge in drug discovery and development [5–15]. Drug side effects cause substantial clinical and economic burdens. The existing computational methods used to predict the side effects of drugs assume that similar drugs have comparable properties in terms of chemical and biological characteristics, such as their structures and targets. However, a literature review revealed that few studies have attempted to predict side effects of each candidate target in the early stage of drug discovery processes. In recent years, applications of computational systems biology have enabled the modelling and analysis of metabolic pathways, regulatory networks and signal transduction networks to better understand cellular behaviour [5,6]. Tumour initiation and progression require the metabolic reprogramming of cancer cells. Cancer cells autonomously alter their metabolic flux through various metabolic pathways; such pathways include aerobic glycolysis, reduced oxidative phosphorylation and the increased generation of biosynthetic intermediates that are required for cell growth and proliferation [16–18]. Numerous studies have employed metabolic reprogramming to understand cancer-specific metabolic networks in order to facilitate drug discovery for cancer treatment [19–32].

Constraint-based modelling approaches entail the use of context-specific genome-scale metabolic networks to identify anti-cancer targets [19–30,32–38]. However, most computer-aided screening methods apply synthetic lethality as a therapeutic indication and ignore side effects during the identification of drug targets. To the best of our knowledge, few studies have attempted to predict side effects of the identified potential targets in the early stage of drug discovery processes. Metabolic flux distributions can alter when normal cells are perturbed due to dysregulation or exerting drug treatment. Metabolic perturbation can be applied to evaluate the influence of the perturbed cell relative to its normal cell counterpart and derives as a measure of side effect for each identified target. The present study used the metabolic perturbation of normal cells relative to the normal template to evaluate side effects for each identified target. A study introduced a fuzzy multi-objective optimization framework for identifying anti-cancer targets (IACT) in order to identify targets that not only increase the mortality of cancer cells but also minimize side effects due to treatment [39]. In addition, the existence and limitations of the IACT framework were theoretically verified [40]. The IACT framework uses RNA-seq expressions of colon cancer cells and their normal counterparts to reconstruct context-specific genome-scale metabolic networks. However, this framework does not include these RNA-seq expressions in computations for inner optimization problems to obtain uniform flux distributions (UFDs). The present study extended the IACT framework to account for RNA-seq information in inner optimization problems in order to yield uniform flux patterns for treated and perturbed cells; these were then applied to identify anti-cancer targets for treating HNSCC.

## 2. Materials and methods

### 2.1. Tissue-specific genome-scale metabolic models

We combined a human metabolic network (Recon3D) [41] with RNA-seq expression data obtained from The Cancer Genome Atlas (TCGA) database [42] to reconstruct population-based tissue-specific genome-scale metabolic models (GSMMs) for HNSCC and healthy counterpart tissues. RNA-seq data of 44 healthy tissue samples with FPKM-UQ normalized expression levels and 500 cancer tissue samples were downloaded from the TCGA database [42] and normalized through quantile normalization; the mean, confidence interval and coefficient of dispersion were then calculated for each gene. These data were used to evaluate supportive genes and classify them into four groups (high, medium, low and not detected). The four gene groups were used to compute the confidence score for each reaction based on the gene–protein-reaction (GPR) associations in Recon3D [41] and classify all reactions into four groups (high, medium, negative and other confidence). The CORDA algorithm [43] used a cost optimization reaction dependency assessment for each confidence reaction to build concise, but not minimalistic, tissue-specific metabolic models for HNSCC and healthy counterpart cells. We developed an integrated toolbox that comprised the aforementioned model reconstruction procedures and to automatically build stoichiometric and GPR models in a computer language of the general algebraic modelling system (https://www.gams.com/) to discover anti-cancer targets with few side effects.

**Figure 1.**
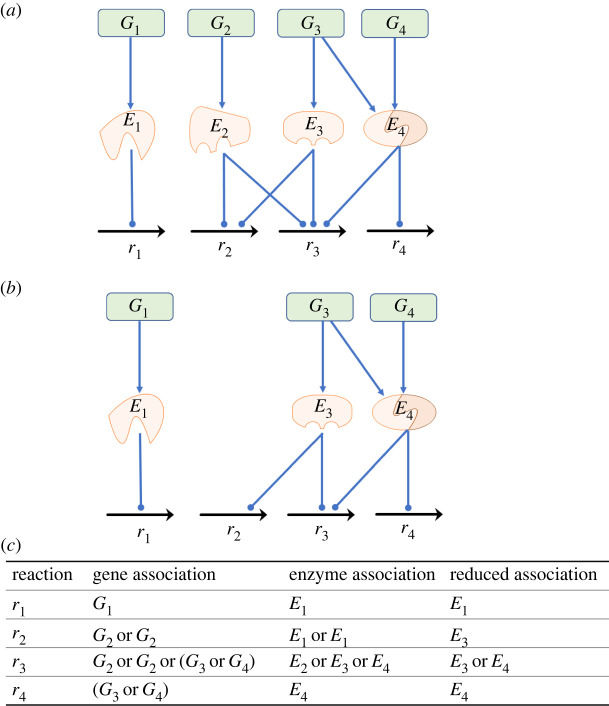
Simplified GPR model in a network. (*a*) Four reactions and their GPR associations. (*b*) Simplified GPR model obtained by deleting redundant associations. (*c*) Boolean rules of GPR models. The enzyme *E_3_* is a redundant enzyme that is identical to *E_2_* and catalyses the same reactions: *r_2_* and *r_3_*. The reaction *r_3_* is catalysed by the isozymes *E_2_* or *E_4_*. *E_1_* is encoded by *G_1_* to regulate *r_1_*, and the promiscuous enzymes *E_2_* and *E_4_* are encoded by *G_2_* and a complex of *G_4_* and *G_5_*, respectively.

Genome-scale reconstructions provide a mechanistic link between genotype and phenotype. Stoichiometric models represent the material balance of metabolites in cells. GPR associations are typically implemented as Boolean rules, which link metabolic reactions in the stoichiometric models to gene-encoded enzymes in cells. A GPR model generally comprises one-to-one enzymes that catalyse a reaction, and promiscuous enzymes that modulate multiple reactions. Therefore, a reaction can be catalysed by one-to-one enzymes or by isozymes. A simple enzyme is encoded by a single gene, whereas a complex enzyme is encoded by at least two genes. In this study, a simplified network (figure 1) was used to describe the GPR associations. In this network, reactions *r_2_* and *r_3_* are catalysed by enzyme *E_2_* or *E_3_* (figure 1*a*); thus, the regulation of both enzymes is identical. We deleted the redundant enzymes in the GSMMs to represent the reduced GPR association (figure 1*b*), thereby avoiding surplus computations in the evolutionary optimization procedures.

### 2.2. Anti-cancer target discovery problem

During discovery and development, drugs generally fail in the clinical trials for two reasons: they are less efficacious or are demonstrated to have serious side effects during initial trials. This study presents an anti-cancer target discovery (ACTD) platform for identifying potential candidate targets with few side effects. The platform integrates RNA-seq expressions for cancer cells and their healthy counterparts into the IACT framework [39,40] to yield uniform flux patterns for treated and perturbed cells. We formulated this extended platform as a fuzzy hierarchical optimization problem. We considered four fuzzy goals in the outer optimization problem in the ACTD platform; we established these goals to account for cancer (CA) cell mortality, healthy (HT) cell viability, perturbation effects and side effects. We directly used fluxes and metabolite flow rates to evaluate the fuzzy objectives. This approach differs from that of the IACT framework, which uses logarithmic fold changes that may have numerical inaccuracies if a value is close to zero. Moreover, in the ACTD platform, the goal targeting CA cell mortality is equivalent to the minimization of not only the cell growth rate but also ATP production rate. The four goals are described as follows:

2.1  The first goal is to measure the mortality of CA cells for treatment. {Fuzzy minimization of the cell growth rate and ATP production rate of treated CA cells:minδ,z~ vbiomassTR≈0,minδ,z~ vATPTR≈0 The second goal is to evaluate cell viability of HT cells due to perturbation. {Fuzzy minimization of the cell growth rate of perturbed HT cells:minδ,z~ vbiomassPB≈0Fuzzy maximization of the ATP production rate of pertubed HT cells:maxδ,z~ vATPPB≈vATPmax The third goal is to measure flux pattern of perturbed HT cells relative to  the CA template. {Fuzzy dissimilarity of fluxes and metabolite flow rates of perturbed HT cells relative to the CA template:dissimilarityδ,z~vjPB⧸≈vjCA,dissimilarityδ,z~rmPB⧸≈rmCA The fourth goal is to measure flux pattern of perturbed HT cells relative to  the HT template. {Fuzzy similarity of fluxes and metabolite flow rates of perturbed HT cells relative to the HT template:similarity~δ,z⁡vjPB≈vjHT,similarityδ,z~ rmPB≈rmHT

Here $\tilde{\min}$ and $\tilde{\max}$ denote fuzzy minimization and maximization, respectively. Moreover, $\tilde{\mathrm{dissimilarity}}$ denotes fuzzy dissimilarity and is used to evaluate the disparity between the fluxes $v_{j}^{PB}$ and metabolite flow rates $r_{m}^{PB}$ for perturbed HT cells relative to the CA template. A significant disparity indicates that the flux distributions and metabolite flow rates of the perturbed HT cells differ considerably from those of the CA template, implying that the perturbation of the HT cells may not lead to tumorigenesis due to treatment. In addition, $\tilde{\mathrm{similarity}}$ denotes fuzzy similarity and is equivalent to a fuzzy equal operator [44]; it is used to evaluate metabolic deviations between the fluxes and metabolite flow rates of the perturbed HT cells relative to the HT template. Smaller metabolic deviations imply lower side effects for a treatment.

The pioneering fuzzy optimization is introduced by Bellman & Zadeh [45] and reveals that fuzzy objectives and/or the constraints constitute classes of alternatives whose boundaries are not sharply defined, and discriminates from a traditional (crisp) optimization. Accordingly, fuzzy minimization (maximization) indicates that the minimum (maximum) objective is less (higher) than its aspiration as possible, and not substantially to fulfil the optimal result completely. Fuzzy similarity is different to a least square error estimation used in a crisp optimization to account for how closeness of the predicted values to the standard level. Furthermore, a crisp optimization method is unable to evaluate a dissimilarity criterion. The present study introduces fuzzy dissimilarity to measure how discrepancy between the predicted values and their standard levels.

Regarding the inner optimization problem, we used constraint-based models of CA and HT cells to calculate the metabolic distributions of treated CA cells and perturbed HT cells, respectively. We reconstructed the tissue-specific GSMMs by using the RNA-seq expression levels of the CA and HT cells to classify metabolic reactions into four groups: high, medium, negative and other confidence, respectively. The weighting factors for the four reaction classes were used to solve the UFD problem to derive a uniform distribution. The inner optimization problem is described as follows:

2.2 $$\left\{ \begin{array}{l} \mathrm{Treated}\;\mathrm{CA}\;\mathrm{model}:\;\\ \mathrm{FBA}\;\mathrm{problem}:\;\;\;\;\;\mathrm{UFD}\;\mathrm{problem}:\;\\ \left\{ \begin{array}{l} \underset{v_{\;f/b}}{\max}v_{\mathrm{biomass}}\;\\ \mathrm{subject}\;\mathrm{to}\;\;\\ N^{CA}(v_{f}-v_{b})=0\;\\ v_{\;f/b,i}^{LB,TR}\leq v_{\;f/b,i}\leq v_{\;f/b,i}^{UB,TR},i\in \Omega^{TR}\;\\ v_{\;f/b,j}^{LB}\leq v_{\;f/b,j}\leq v_{\;f/b,j}^{UB},j\notin \Omega^{TR}\; \end{array}\right.\left\{ \begin{array}{l} \underset{v_{\;f/b}}{\min}\sum_{k\in \Omega^{Int}}c_{k}^{CA}({\left(v_{\;f,k}\right)}^{2}+{\left(v_{b,k}\right)}^{2})\;\\ \mathrm{subject}\;\mathrm{to}\;\;\\ N^{CA}(v_{f}-v_{b})=0\;\\ v_{\;f/b,i}^{LB,TR}\leq v_{\;f/b,i}\leq v_{\;f/b,i}^{UB,TR},i\in \Omega^{TR}\;\\ v_{\;f/b,j}^{LB}\leq v_{\;f/b,j}\leq v_{\;f/b,j}^{UB},j\notin \Omega^{TR}\;\\ v_{\mathrm{biomass}}\geq v_{\mathrm{biomass},CA}^{*}\; \end{array}\right.\;\\ \;\\ \mathrm{Perturbed}\;\mathrm{HT}\;\mathrm{model}:\;\\ \mathrm{FBA}\;\mathrm{problem}:\;\;\;\;\;\mathrm{UFD}\;\mathrm{problem}:\;\\ \left\{ \begin{array}{l} \underset{v_{\;f/b}}{\max}v_{\mathrm{ATP}}\;\\ \mathrm{subject}\;\mathrm{to}\;\;\\ N^{HT}(v_{f}-v_{b})=0\;\\ v_{\;f/b,i}^{LB,TR}\leq v_{\;f/b,i}\leq v_{\;f/b,i}^{UB,TR},i\in \Omega^{TR}\;\\ v_{\;f/b,j}^{LB}\leq v_{\;f/b,j}\leq v_{\;f/b,j}^{UB},j\notin \Omega^{TR}\; \end{array}\right.\left\{ \begin{array}{l} \underset{v_{\;f/b}}{\min}\sum_{k\in \Omega^{Int}}c_{k}^{HT}({\left(v_{\;f,k}\right)}^{2}+{\left(v_{b,k}\right)}^{2})\;\\ \mathrm{subject}\;\mathrm{to}\;\;\\ N^{HT}(v_{f}-v_{b})=0\;\\ v_{\;f/b,i}^{LB,TR}\leq v_{\;f/b,i}\leq v_{\;f/b,i}^{UB,TR},i\in \Omega^{TR}\;\\ v_{\;f/b,j}^{LB}\leq v_{\;f/b,j}\leq v_{\;f/b,j}^{UB},j\notin \Omega^{TR}\;\\ v_{ATP}\geq v_{\mathrm{ATP},HT}^{*}\; \end{array}\right.\; \end{array}\right.,$$

where **v***_f/b_* is the forward–backward flux vector of reactions; **N***^CA^* and **N***^HT^* are stoichiometric matrices for tissue-specific CA and HT cells, respectively, and are reconstructed from those presented in the previous section; $v_{\;f/b,j}^{LB}$ and $v_{\;f/b,j}^{UB}$ are the positive lower bound (LB) and upper bound (UB) of the *j*th forward–backward flux, respectively; and Ω*^TR^* is the set of reactions for CA and HT models. The weighting factors $c_{k}^{CA}\mathrm{and}\;c_{k}^{HT}$ depended on the classified groups and can be assigned as follows:

2.3 $$c_{k}^{CA/HT}=\left\{ \begin{array}{c} \frac{1}{4},k\in \mathrm{high}\;\mathrm{confidence}\;\\ \frac{1}{2},k\in \mathrm{medium}\;\mathrm{confidence}\;\\ \frac{3}{4},k\in \mathrm{negative}\;\mathrm{confidence}\;\\ 1,\;k\in \mathrm{other}\;\mathrm{confidence}\;\mathrm{or}\;\mathrm{non}\text{-}\mathrm{gene}\text{-}\mathrm{expression}\; \end{array}\right.$$

For a high confidence reaction, we can set the smallest weighting factor to obtain a higher flux value from the UFD problem. $v_{\;f/b,i}^{LB,TR}$ and $v_{\;f/b,i}^{UB,TR}$ denote the LB and UB of the regulated forward–backward fluxes depended on gene- or metabolite-centric approach for activation. The regulation bounds for the gene-centric approach can be expressed as follows:

2.4  Regulated bounds for zith active gene/enzyme: Up-regulation: {(1−δ)v f,ibasal+δv f,iUB≤v f,i≤ v f,iUBvb,iLB≤vb,i≤(1−δ)vb,ibasal+δvb,iLB; zi∈ΩTR Down-regulation: {v f,iLB≤v f,i≤(1−δ)v f,ibasal+δv f,iLB(1−δ)vb,ibasal+δvb,iUB≤vb,i≤vb,iUB; zi∈ΩTR∖ΩIZ {(1−ε)v f,ibasal≤v f,i≤(1+ε)v f,ibasal(1−ε)vb,ibasal≤vb,i≤(1+ε)vb,ibasal; zi∈ΩTR∩ΩIZ Knockout: {v f,i=0vb,i=0; zi∈ΩTR∖ΩIZ {(1−ε)v f,ibasal≤v f,i≤(1+ε)v f,ibasal(1−ε)vb,ibasal≤vb,i≤(1+ε)vb,ibasal; zi∈ΩTR∩ΩIZ,

where $v_{\;f,i}^{\mathrm{basal}}$ and $v_{b,i}^{\mathrm{basal}}$ are the basal value of the *i*th forward–backward flux obtained from XA and HT templates; *Ω^IZ^* is the set of reactions regulated by isozymes determined using the GPR associations, and *δ* is the modulation parameter determined by a nested hybrid differential evolution (NHDE) algorithm [39,40,46–48]. A reaction catalysed by isozymes remains around its basal level; thus, we set the flux ratio *ε* to 0.03 in this study to restrict the flux value. Metabolite-centric regulators modulate the synthesis reactions of the active metabolites. The LBs and UBs of modulated reactions for the *i*th active metabolite are restricted as follows:

2.5  Regulated bounds for the zith active metabolite: Up-regulation: {(1−δ)v f,jbasal+δv f,jUB≤v f,j≤ v f,jUB;j∈Nij>0 and j∈Ωrxn(1−δ)vb,jbasal+δvb,jUB≤vb,j≤ vb,jUB;j∈Nij<0 and j∈Ωrev Down-regulation: {v f,jLB≤v f,j≤(1−δ)v f,jbasal+δv f,jLB;j∈Nij>0 and j∈Ωrxnvb,jLB≤vb,j≤(1−δ)vb,jbasal+δvb,jLB;j∈Nij<0  and j∈Ωrev Knockout: {v f,j=0;j∈Nij>0and j∈Ωrxnvb,j=0; j∈Nij<0 and j∈Ωrev,

where *N_ij_* is the stoichiometric coefficient of the *i*th metabolite and the *j*th reaction.

### 2.3. Maximizing decision-making problem

**Figure 2.**
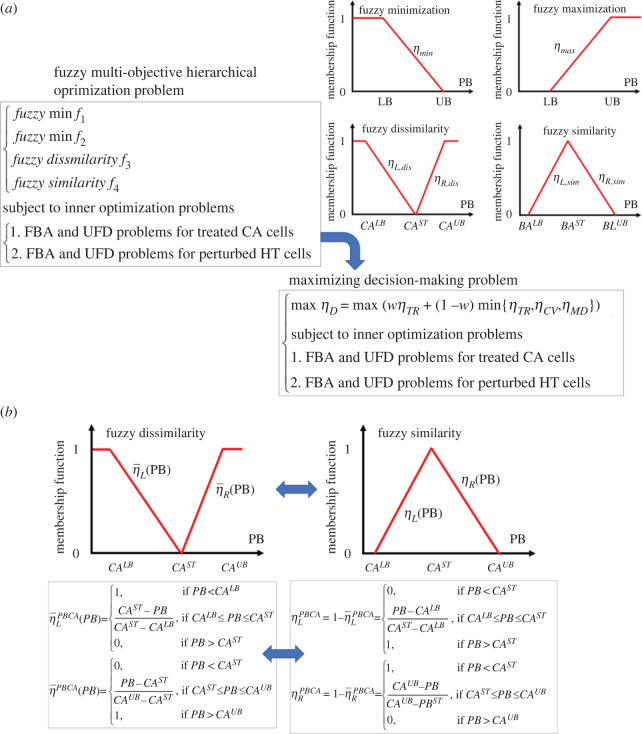
Description of the mathematical strategy for solving a fuzzy multi-objective hierarchical optimization problem. (*a*) Transformation of a fuzzy multi-objective hierarchical optimization problem into an MDM problem through fuzzy membership functions. (*b*) Two-sided linear membership function for representing fuzzy dissimilarity and its relationship with fuzzy similarity.

The ACTD problem expressed in equations (2.1) and (2.2) can be transformed into a maximizing decision-making (MDM) problem by using fuzzy set theory to derive Pareto solutions (figure 2*a*). Fuzzy minimization and maximization objectives can be assigned membership grades according to their corresponding one-side line membership functions as follows:

2.6 $$\eta_{\min}=\left\{ \begin{array}{c} 1,\;\mathrm{if}\;FV<LB\;\\ \frac{UB-FV}{UB-LB},\;\mathrm{if}\;LB\leq FV\leq UB\;\\ 0,\;\mathrm{if}\;FV>UB\; \end{array}\right.$$

and

2.7 $$\eta_{\max}=\left\{ \begin{array}{c} 0,\;\mathrm{if}\;FV<LB\;\\ \frac{FV-LB}{UB-LB},\;\mathrm{if}\;LB\leq FV\leq UB\;\\ 1,\;\mathrm{if}\;FV>UB\; \end{array}\right.,$$

where *FV* represents the fluxes and metabolite flow rates for the treated CA and perturbed HT cells, which are computed from equation (2.2); moreover, the LBs and UBs can be provided by a user in advance. These bounds can be obtained from clinical experimental data (if available). Fuzzy dissimilarity is a complement of fuzzy similarity; therefore, the relationship of both membership functions is convertible (figure 2*b*). A fuzzy similarity grade can be assigned using a two-sided line membership function as follows:

2.8  Left-hand side membership function: ηLPBST={0, if PB<LBPB−LBST−LB, if LB≤PB≤ST1, if PB=ST Right-hand side membership function: ηRPBST={1, if PB=STUB−PBUB−ST, if ST≤PB≤UB0, if PB>UB,

where ST represents the standard level of CA or HT cells and can be obtained from the corresponding templates of the cells. Therefore, the metabolic deviation grades can be derived from the two-sided line membership function as follows:

2.9 $$\eta_{MD}^{\mathrm{PBST}}=\max\{\min\{\eta_{L}^{\mathrm{PBST}},\eta_{R}^{\mathrm{PBST}},1\},0\}$$

The metabolic deviation grade ranges between 0 and 1. A value of 0 indicates that an objective has not been met at all, whereas a value of 1 indicates a completely fulfilled objective.

In the ACTD problem, the first goal is to consider the mortality of the treated CA cells; the membership grades $\eta_{\mathrm{biomass}}^{TR}\mathrm{and}\;\eta_{\mathrm{ATP}}^{TR}$ can be derived using equation (2.6). We define a mean–min operator to evaluate the cell mortality grade $\eta_{TR}$ as follows:

2.10 $$\eta_{TR}=\frac{\left(1/2(\eta_{\mathrm{biomass}}^{TR}+\eta_{\mathrm{ATP}}^{TR})+\min\{\eta_{\mathrm{biomass}}^{TR},\eta_{\mathrm{ATP}}^{TR}\}\right)}{2}.$$

The operator can discriminate cases with identical means, as demonstrated by the numerical example in table 1. The second objective in the ACTD problem is to evaluate cell viability; the cell viability grade $\eta_{CV}$ can be derived through a similar procedure to that indicated in equation (2.10). The third and fourth objectives are to evaluate metabolic deviation grades of perturbed HT cells from the CA and HT templates. The fluxes *v_f,j_* and *v_b,j_* of the perturbed cells can first be obtained from equation (2.2) and then used to compute the metabolite flow rate *r_m_* of the *m*th metabolite as follows:

2.11 $$r_{m}=\sum_{i\in \Omega^{c}}\left(\sum_{N_{ij}>0,j}N_{ij}v_{\;f,j}-\sum_{N_{ij}<0,j}N_{ij}v_{b,j}\right),m\in \Omega^{m},$$

where Ω*^c^* denotes the set of species located in different compartments and *N_ij_* denotes stoichiometric coefficients. Both fluxes and metabolite flow rates can be used to compute two-sided membership grades, as indicated in equation (2.8), and to obtain fuzzy dissimilarity membership grades $\left(\eta_{f}^{PBCA}\;\mathrm{and}\;\eta_{m}^{PBCA}\right)$ and fuzzy similarity membership grades $\left(\eta_{f}^{PBBL}\;\mathrm{and}\;\eta_{m}^{PBBL}\right)$ for metabolic alterations for perturbed cells from the CA and HT templates, respectively. Accordingly, the overall metabolic deviation grade can be derived as follows:

2.12 $$\eta_{MD}=\frac{\eta_{f}^{PBCA}+\eta_{m}^{PBCA}+\eta_{f}^{PBBL}+\eta_{m}^{PBBL}}{4}+\frac{\min\{\eta_{f}^{PBCA},\eta_{m}^{PBCA},\eta_{f}^{PBBL},\eta_{m}^{PBBL}\}}{2}.$$

The metabolic deviation grade ranges between 0 and 1. A higher metabolic deviation grade implies a smaller degree of metabolic perturbation due to treatment, suggesting that the treatment has fewer side effects.

**Table 1.** Numerical example demonstrating mean, min and mean–min evaluations.

**case**	$\eta_{\mathrm{biomass}}^{TR}$	$\eta_{\mathrm{ATP}}^{TR}$	$(\eta_{\mathrm{biomass}}^{TR}+\eta_{\mathrm{ATP}}^{TR})/2$	$\min\{\eta_{\mathrm{biomass}}^{TR},\eta_{\mathrm{ATP}}^{TR}\}$	$\eta_{TR}$
**1**	1	0	0.5	0	0.25
**2**	0.5	0.5	0.5	0.5	0.5
**3**	0.4	0.6	0.5	0.4	0.45

For expansions for all the abbreviated term in all tables, refer to Abbreviations.

The ACTD problem in equations (2.1) and (2.2) can be transformed into an MDM problem through the aforementioned membership functions. The optimality and limitation of the transformation have been proved in Wang *et al*. [40]. The MDM problem is expressed as follows:

2.13 $$\left\{ \begin{array}{c} \underset{\delta ,z}{\max}\eta_{D}=\underset{\delta ,z}{\max}\left(\eta_{TR}+\min\{\eta_{TR},\eta_{CV},\eta_{MD}\}\right)/2\;\\ \mathrm{subject}\;\mathrm{to}\;\mathrm{inner}\;\mathrm{optimization}\;\mathrm{problems}\;\\ 1.\;\mathrm{FBA}\;\mathrm{and}\;\mathrm{UFD}\;\mathrm{problems}\;\mathrm{for}\;\mathrm{treated}\;\mathrm{CA}\;\mathrm{cells}\;\\ 2.\;\mathrm{FBA}\;\mathrm{and}\;\mathrm{UFD}\;\mathrm{problems}\;\mathrm{for}\;\mathrm{perturbed}\;\mathrm{HT}\;\mathrm{cells},\; \end{array}\right.$$

where the decision objective *η_D_* is a hierarchical criterion. This criterion states that the mortality grade *η_TR_* for treated CA cells is the first priority in the decision-making problem; moreover, if either the cell viability or metabolic deviation grade is less than the mortality grade, then one of the lowest grades in the set of {*η_TR_*, *η_CV_*, *η_MD_*} is the second priority for decision-making. The decision-making problem in equation (2.13) is a mixed-integer optimization problem that involves linear and quadratic programming problems in its inner loop. It is a high-dimensional NP-hard problem that cannot be solved by available commercial software [49,50]. We employed the NHDE algorithm to solve the MDM problem. The NHDE algorithm is a parallel direct search procedure, which is an extended version based on hybrid differential evolution [51]. The detailed computational procedures are provided in the electronic supplementary material [52].

## 3. Results and discussion

**Figure 3.**
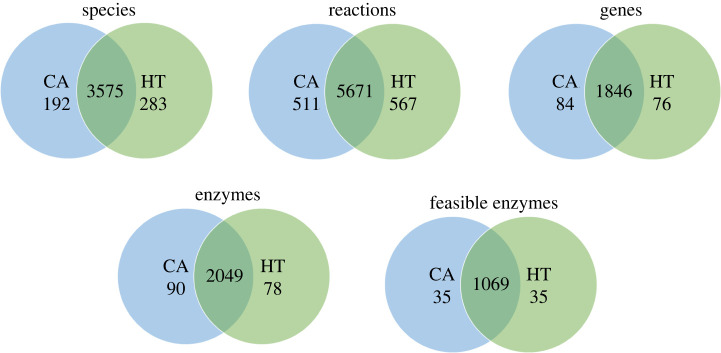
The comparison of metabolic network data between the HT and CA models was reconstructed through the CORDA algorithm. Statistics of the reconstructed metabolic models for CA and HT cells are also indicated. The numbers listed in the overlapping regions denote the number of identical species, reactions, genes, enzymes and feasible enzymes in the HT and CA models. Feasible enzymes were separated from redundant enzymes through GPR association reduction.

We reconstructed tissue-specific GSMMs for CA and HT cells, that are provided in the electronic supplementary material [52], and figure 3 illustrates the corresponding numbers of species, reactions, genes and encoded enzymes. As indicated by the overlapping regions in figure 3, both models shared numerous similarities in terms of species, reactions, genes and enzymes. Feasible enzymes were separated from redundant enzymes through the reduced GPR associations and were used as candidate variables in equation (2.13) to solve the MDM problem, thus avoiding trivial optimization searches. The iMAT algorithm [53] was also used to reconstruct tissue-specific GSMMs for CA and HT cells, and the corresponding numbers of species, reactions, genes and encoded enzymes are listed in the electronic supplementary material [52] for comparison. The CORDA algorithm [43] is not to keep the reconstructed model as concise as possible. As a result, these numbers are greater than those reconstructed by the iMAT algorithm.

### 3.1. Identifying anti-cancer target genes

We first applied the NHDE algorithm [39,40,46–48] to solve the MDM problem in order to identify optimal anti-cancer target genes; we determined a set of 21 one-target genes with the highest hierarchical fitness among 1104 feasible candidates (table 2). GSMMs for CA and HT cells reconstructed by the iMAT algorithm were also used for the inner optimization problem in equation (2.2) to solve the MDM problem. The optimal anti-cancer target genes were identical to those of the reconstructed models using the CORDA algorithm (see the electronic supplementary material [52]). The cell mortality and cell viability grades obtained using the models reconstructed by CORDA were less than those of the models reconstructed by iMAT, but achieved higher metabolic deviation grade.

**Table 2.** Top 21 one-target genes obtained using the ACTD framework. *η_TR_* and *η_MD_* denote the cell mortality grade for treated CA cells and the overall metabolic deviation grade for perturbed HT cells for CA and HT templates, respectively. ‘N/D’ is the cell death number (N) divided by the total number of head and neck CA cells (D) used for the test from DepMap. ‘no. drugs’ denotes the number of drugs retrieved from DrugBank that modulate each gene.

gene	*η_TR_*	*η_CV_*	*η_MD_*	N/D	no. drugs
*RPIA*	0.668	0.751	0.682	16/68	1
*DHODH*	0.668	0.751	0.599	9/68	26
*PGS1*	0.668	0.751	0.581	65/68	–
*CAD*	0.668	0.751	0.566	18/68	3
*SLC7A6*	0.668	0.751	0.558	0/68	1
*SPTLC3*	0.668	0.751	0.551	0/68	1
*KDSR*	0.668	0.751	0.551	10/68	–
*CRLS1*	0.668	0.751	0.531	48/68	–
*UMPS*	0.668	0.751	0.530	16/68	2
*ATIC*	0.668	0.751	0.517	6/68	9
*PFAS*	0.668	0.751	0.513	8/68	2
*TYMS*	0.668	0.751	0.505	39/68	24
*PAICS*	0.668	0.751	0.498	18/68	1
*ACACA*	0.668	0.751	0.488	31/68	1
*PPAT*	0.668	0.751	0.484	36/68	4
*ADSS2*	0.668	0.751	0.459	20/68	2
*ADSL*	0.668	0.751	0.448	38/68	2
*DHFR*	0.654	0.741	0.457	54/68	1
*SLC2A13*	0.621	0.716	0.208	0/68	1
*SLC5A3*	0.564	0.673	0.211	1/68	1
*CEPT1*	0.372	0.529	0.643	38/68	2

A survey of the Cancer Dependency Map (DepMap, https://depmap.org/portal/) revealed that most of the identified target genes were compatible to HNC cell lines from DepMap and that these genes could engender a high percentage of cell death when knocked out, except for *SLC7A6*, *SPTLC3* and *SLC2A13*. The computation results (table 2) revealed that targeting all identified genes for treatment could lead to complete cell death; however, the ATP production rates differed between the targets. Thus, targeting most of these genes for treatment, except for *DHFR*, *SLC2A13*, *SLC5A3* and *CEPT1*, could lead to cell mortality grade of 0.668. Similarly, targeting most of the identified genes for the treatment of CA cells and perturbed HT cells could lead to cell viability grades of 0.751. Among the identified one-target genes (table 2), we observed that *RIPA* knockout yielded the highest metabolic deviation grade (0.682). The metabolic deviation grade is used to evaluate metabolic perturbation for fluxes and metabolite flow rates from the HT and CA templates; thus, a higher metabolic deviation grade implies a smaller metabolic perturbation due to treatment; that is, the treatment would have fewer side effects.

**Figure 4.**
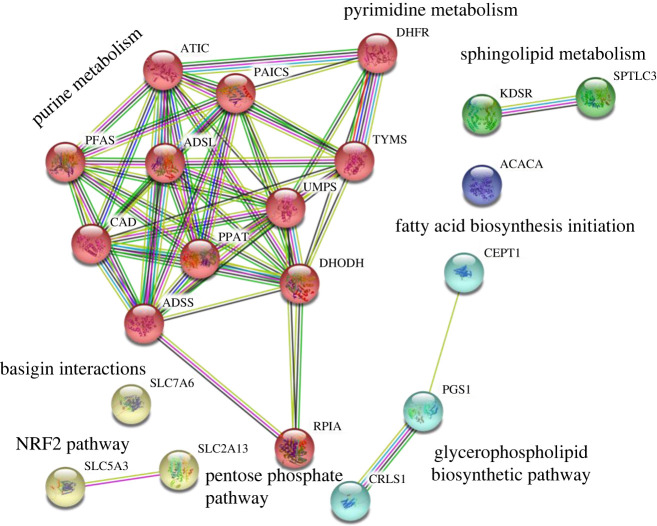
PPIs of the identified anti-cancer enzymes. MCL clustering in the STRING database was applied to classify one-target enzymes into four classes. Enzymes involved in purine metabolism are outlined as follows: *ATIC, PAICS, TYMS, PFAS, ADSL, UMPS, CAD, PPAT, DHODH* and *ADSS*. Enzymes involved in pyrimidine metabolism are outlined as follows: *DHFR, TYMS, UMPS, CAD, PPAT* and *DHODH*. Some enzymes overlap (i.e. they are involved in both metabolisms).

The protein–protein interaction (PPI) networks of the identified 21 one-target genes were investigated using STRING (https://string-db.org/), as presented in figure 4. Markov clustering in STRING was used to classify the PPI network into five classes, which are presented as colour nodes in figure 4. The first class (red nodes) comprised 12 enzymes involved in purine and pyrimidine metabolism and in the pentose phosphate pathway. The enzymes in the second to fifth classes were determined to be involved in the glycerophospholipid biosynthetic pathway, sphingolipid metabolism, NRF2 pathway and basigin interactions. We also performed a brute-force enumerative search to evaluate 1104 feasible enzymes individually to validate the computational results presented in table 2. However, targeting genes such as *HMGCR* and *FDFT1* involved in the statin-induced mevalonate pathway could not eliminate CA cell growth. These results are not consistent with those derived using the IACT framework for IACTs for colon cancer treatment [35].

The ACTD platform can be used to identify multi-target combinations for treating HNSCC. An enumerative search is a time-consuming process because it requires the use of more than 500 000 combinations. Accordingly, we applied the NHDE algorithm by using two groups of candidates to identify multiple targets. The first candidate group comprised the 21 targets earlier (table 2), and the second group comprised other candidate genes from the feasible enzymes. This strategy substantially reduced the computational time and reduced the search space to only 22 743 (21 × 1083) possible combinations between the two candidate groups. We performed a series of computations to obtain a set of two-target combinations. Numerous two-target combinations were identified, and table 3 presents the optimal two-target combinations with cell viability grades greater than 0.75; other combinations are listed in the electronic supplementary material [52]. We observed that the combination of phosphoglycerate kinase 1 (*PGK1*) with each target enzyme (*CAD*, *PAICS* and *DHODH*) in the pyrimidine metabolic pathway improved the cell viability grade by more than 22% (*η_CV_* > 0.92) but reduced the metabolic deviation grade by approximately 6% (*η_MD_* < 0.526) compared with the use of the single-target counterparts of these enzymes (table 2). Among the targets, *ATIC* was determined to participate in purine metabolism, and its combination with glutathione reductase (*GSR*) could increase the cell viability grade by more than 26% and the metabolic deviation grade by 23% (*η_MD_* = 0.636).

**Table 3.** Top 30 two-target combinations obtained using the ACTD framework. *TR* and *MD* denote the cell mortality grade for treated CA cells and the overall metabolic deviation grade for perturbed HT cells to the CA and HT templates, respectively. ‘N/D’ represents the cell death number (N) divided by the total number of head and neck CA cells (D) used for the test from DepMap. ‘no. drugs’ denotes the number of drugs retrieved from DrugBank that modulate each gene. UQCR: commonly referred to as complex III. Cytochrome is a heme-containing subunit of the cytochrome b-c1 complex. COX: Complex of cytochrome c oxidase.

target combination	*η_TR_*	*η_CV_*	*η_MD_*	N/D	no. drugs
(*CAD*, *PGK1*)	0.914	0.935	0.526	(18/68, 65/68)	(3, 5)
(*ATIC*, *GSR*)	0.912	0.934	0.636	(6/68, 0/68)	(9, 18)
(*PAICS*, *PGK1*)	0.902	0.926	0.470	(18/68, 65/68)	(1, 5)
(*DHODH*, *PGK1*)	0.893	0.920	0.522	(9/68, 65/68)	(26, 5)
(*TAT*, CYC-COX)	0.863	0.897	0.584	(0/68, 4/68)	(4, 7)
(*KDSR*, CYC-COX)	0.853	0.890	0.604	(10/68, 4/68)	(–, 7)
(*PGS1*, CYC-COX)	0.851	0.888	0.612	(65/68, 4/68)	(–, 7)
(*SLC7A6*, CYC-COX)	0.843	0.882	0.631	(0/68, 4/68)	(1, 7)
(*TYMS*, CYC-COX)	0.835	0.876	0.626	(39/68, 4/68)	(24, 7)
(*PGS1*, CYC-COX)	0.832	0.874	0.628	(65/68, 4/68)	(–, 7)
(*PFAS*, CYC-COX)	0.830	0.873	0.628	(8/68, 4/68)	(2, 7)
(*PAICS*, CYC-COX)	0.830	0.873	0.628	(18/68, 4/68)	(1, 7)
(*PPAT*, CYC-COX)	0.829	0.872	0.629	(36/68, 4/68)	(4, 7)
(*ACACA*, CYC-COX)	0.822	0.867	0.629	(31/68, 4/68)	(1, 7)
(*CAD*, CYC-COX)	0.822	0.867	0.610	(18/68, 4/68)	(3, 7)
(*UMPS*, CYC-COX)	0.818	0.863	0.613	(16/68, 4/68)	(2, 7)
(*ATIC*, CYC-COX)	0.815	0.862	0.630	(6/68, 4/68)	(9, 7)
(*CRLS1*, CYC-COX)	0.815	0.861	0.630	(48/68, 4/68)	(–, 7)
(*DHODH*, CYC-COX)	0.814	0.861	0.621	(9/68, 4/68)	(26, 7)
(*UMPS*, *HMGCR*)	0.668	0.751	0.701	(16/68, 65/68)	(2, 20)
(*CAD*, *PC*)	0.671	0.754	0.694	(18/68, 61/68)	(3, 3)
(*CEPT1*, *DHODH*)	0.668	0.751	0.691	(38/68, 9/68)	(2, 26)
(*CAD*, *HMGCR*)	0.668	0.751	0.689	(18/68, 65/68)	(3, 20)
(*PAICS*, *PRDX6*)	0.668	0.751	0.680	(18/68, 1/68)	(1, 2)
(*SLC5A3*, *TYMS*)	0.667	0.750	0.679	(1/68, 39/68)	(1, 24)
(*CEPT1*, *ATIC*)	0.668	0.751	0.678	(38/68, 6/68)	(2, 9)

The cytochrome complex (abbreviated as CYC-COX) contains multiple ubiquinol–cytochrome c oxidoreductase and cytochrome c oxidase subunits. It is part of the mitochondrial respiratory chain and plays a key role in oxidative phosphorylation. We could not eliminate CA cell growth through CYC-COX downregulation only. However, the combination of CYC-COX with any of the 21 single-target enzymes (table 1) could achieve cell viability and metabolic deviation grades of more than 0.86 and 0.61, respectively. This metabolic deviation grade was noted to be higher than those derived for the *PGK1* combinations, indicating that CYC-COX combination treatments would have fewer side effects. Additionally, *HMGCR* knockdown could not block CA cell growth. However, the two-target combinations of (*UMPS*, *HMGCR*) and (*CAD*, *HMGCR*) could increase the metabolic deviation grade by more than 22% without reducing the cell viability grade.

**Figure 5.**
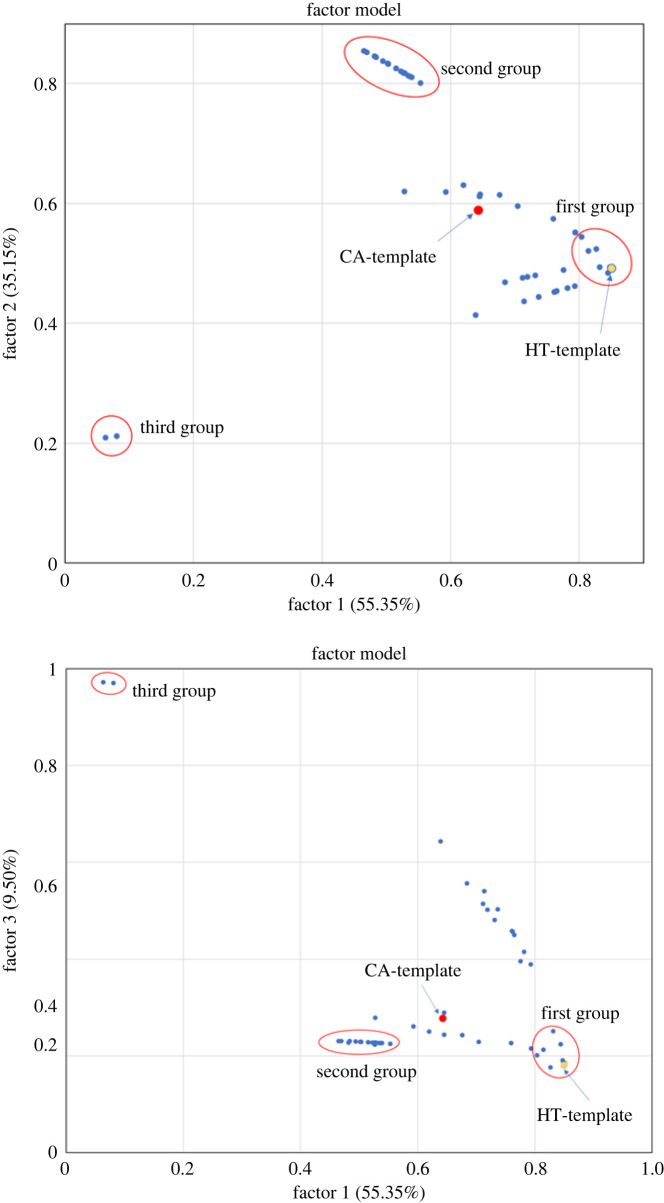
Factor analysis of the metabolite flow rates for one- and two-target anti-cancer enzymes. The first, second and third factors had loadings of more than 0.8; the fourth had smaller loadings.

We composed a set of the perturbed metabolite flow rates derived from the identified targets to perform factor analysis to investigate their flux patterns. The set comprised 2380 metabolite flow rates and 88 cases, including the CA template, HT template and perturbed cases (see the electronic supplementary material [52]). We conducted an iterated principal factor analysis by using the orthogonal varimax rotation method in SAS software (https://www.sas.com/) to analyse the data; the analysis yielded three factors and their corresponding loadings (see electronic supplementary material [52]). The key concept of factor analysis is that multiple observed variables have similar response patterns because they are all associated with a latent (i.e. not directly measured) variable. Our analysis revealed that the first-, second- and third-factor loadings (figure 5) were 55.35%, 35.15% and 9.50%, respectively. For each factor, we collected target variables with a loading of more than 0.8 and classified them into three groups (figure 5). The first group comprised the HT template and 41 one-target and two-target combinations with a first factor loading of greater than 0.8. An investigation using GeneCards (https://geneanalytics.genecards.org/) revealed that these target genes mainly participate in the following metabolic pathways: lipid and lipoprotein metabolism, purine and pyrimidine metabolism, and glycosaminoglycan metabolism. The second group comprised 20 two-target combinations; specifically, they comprised combinations of the CYC-COX complex with any one target involved in lipid and lipoprotein metabolism, purine and pyrimidine metabolism, or glycosaminoglycan metabolism. The third group comprised two transporters, namely *SLC2A13* and *SLC5A3*. Targets with a factor loading of less than 0.8 were classified into the ‘other’ group; this group included the CA template and 23 targets. In addition, the flux distributions for all treatments, CA templates and HT templates are presented as a heatmap in the electronic supplementary material [52] to illustrate differences between the treatments. We observed that the flux distributions for each treatment were similar within groups but differed slightly between groups.

### 3.2. Identifying antimetabolites

**Figure 6.**
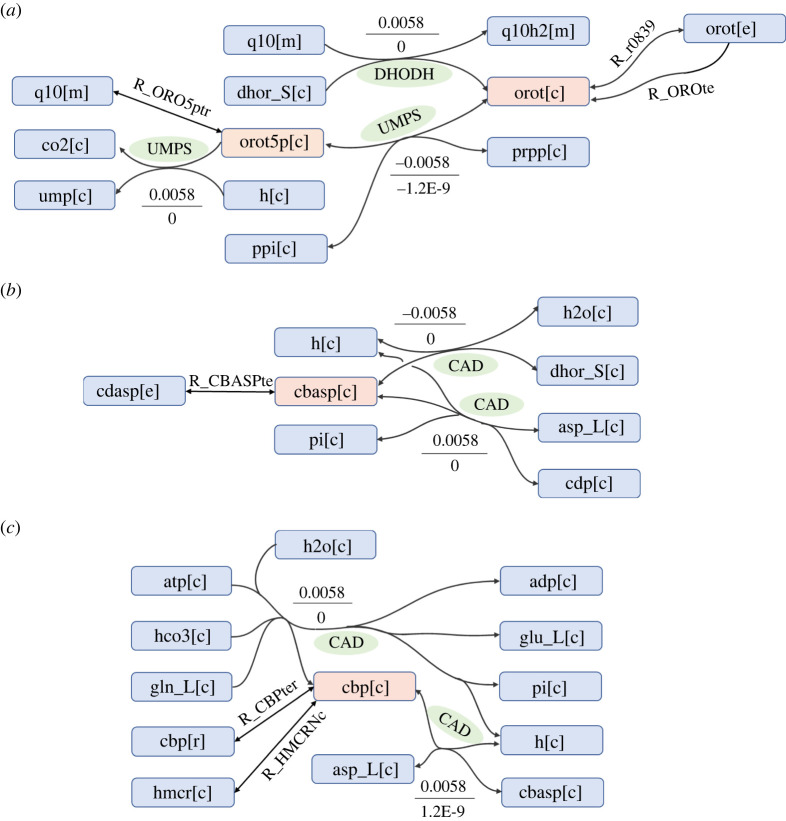
Metabolic pathways for inhibiting the one-target antimetabolites (*a*) orot[c] and orot5p[c], (*b*) cbasp[c] and (*c*) cbp[c]. The flux values for each reaction for CA and HT are presented in the upper (CA) and lower (HT) boxes. Reactions with a name only have a flux value of zero for both templates.

A metabolite-centric approach in the ACTD framework was also used to discover one- and two-target antimetabolites for treating HNSCC. Table 4 presents some of the optimal targets, and the remaining targets are listed in the electronic supplementary material [52]. The one-target antimetabolites orotate (orot), orotidine 5'-phosphate (orot5p), uridine-5'-monophosphate (ump), N-carbamoyl-L-aspartate (cbasp) and carbamoyl phosphate (cbp) are precursors of nucleotides participating in the pyrimidine biosynthetic pathway of DNA synthesis. The inhibition of these metabolites could lead to cell mortality, cell viability and metabolic deviation grades greater than 0.668, 0.751 and 0.537, respectively. Figure 6 illustrates the synthesis of orot from (S)-dihydroorotate (dhor_S) and quinone (q10), which is catalysed by the enzyme *DHODH* (figure 6*a*). Moreover, the enzyme *UMPS* catalyses two reactions that convert orot to orot5p and subsequently to ump (figure 6*a*). The enzyme *CAD* catalyses three reactions (figure 6*b,c*) that convert dhor_S to cbasp and then to cbp. Moreover, *CAD* catalyses the synthesis of L-glutamic acid (glu_L) from L-glutamine (gln_L). The identified antimetabolite treatments were noted to be nearly identical to those identified using the gene-centric approaches (tables 2 and 4). These results indicate that the two-target combinations could improve the metabolic deviation grade by more than 13% compared with one-target treatments; however, increases in the cell viability grade were smaller.

**Table 4.** Membership grades for cancer cell mortality (*η_TR_*), cell viability grade (*η_CV_*) and metabolic deviation (*η_MD_*) for the top 10 antimetabolites of one- and two-target combinations. The symbol ↑ indicates upregulation.

target	*η_TR_*	*η_CV_*	*η_MD_*	pathway
orot[c]	0.668	0.751	0.614	pyrimidine synthesis
orot5p[c]	0.668	0.751	0.594	pyrimidine synthesis
ump[c]	0.682	0.761	0.571	pyrimidine synthesis
cbasp[c]	0.668	0.751	0.537	pyrimidine synthesis
cbp[c]	0.668	0.751	0.537	pyrimidine synthesis
r5p[c]	0.682	0.761	0.521	nucleotide interconversion
gar[c]	0.645	0.734	0.517	purine synthesis
air[c]	0.602	0.701	0.516	purine synthesis
dag_hs[c]	0.668	0.751	0.507	glycerophospholipid metabolism
sphgn[c]	0.662	0.746	0.506	sphingolipid metabolism
(cs_d_deg5[l]↑, gar[c])	0.668	0.751	0.700	(chondroitin sulfate degradation, purine synthesis)
(phpyr[m]↑, trdrd[c])	0.694	0.770	0.698	(phenylalanine metabolism, nucleotide interconversion)
(CE0785[m]↑, 25aics[c])	0.709	0.782	0.694	(fatty acid oxidation, purine synthesis)
(cbp[c], dudp[c])	0.682	0.761	0.692	(pyrimidine synthesis, nucleotide interconversion)
(imp[c], c6crn[c]↑)	0.668	0.751	0.690	(nucleotide interconversion, fatty acid oxidation)
(prpp[c], air[c])	0.682	0.761	0.687	(purine synthesis, purine synthesis)
(asp_L[m], dhor_S[c])	0.671	0.753	0.686	(arginine and proline metabolism, pyrimidine synthesis)
(cbasp[c], dutp[c])	0.682	0.761	0.686	(pyrimidine synthesis, nucleotide interconversion)
(ump[c], dadp[c])	0.682	0.761	0.686	(pyrimidine synthesis, nucleotide interconversion)
(orot[c], r5p[c])	0.682	0.761	0.685	(pyrimidine synthesis, pentose phosphate pathway)

## 4. Conclusion

Computer-aided drug discovery technology has developed rapidly over recent decades. Computational approaches can help screen potential candidate targets to reduce the time and cost of drug development. Current constraint-based modelling methods can discover potential candidate targets for treatments inhibiting CA cell metabolism. Most computer-aided methods use synthetic lethality as a therapeutic criterion for identifying drug targets; however, they do not attempt to predict side effects of each candidate target in the early stage of drug discovery processes. This study developed an ACTD platform and considered four fuzzy goals for treatment: high cell mortality for CA cells, high cell viability for HT cells, minimal treatment effects on HT cells and low side effects for HT cells. Moreover, RNA-seq expression data were applied to reconstruct tissue-specific GSMMs for CA and HT cells and to improve flux balance models for inner optimization problems to obtain uniform distributions.

We applied the ACTD platform to identify one-target and two-target combinations of anti-cancer enzymes and antimetabolites for HNSCC treatment. For the gene-centric approach, the one- and two-target combinations mostly comprised targets participating in purine and pyrimidine metabolism, the pentose phosphate pathway, the glycerophospholipid biosynthetic pathway, sphingolipid metabolism, the NRF2 pathway and basigin interactions. By using DrugBank (https://go.drugbank.com/), we found that many of the identified targets could be modulated by approved drugs; thus, these drugs are potential candidates for repurposing against HNSCC. For the metabolite approach, we identified antimetabolite that was nearly identical to those obtained using the gene-centric approach. *HMGCR* knockdown could not block HNSCC growth. However, the two-target combinations of (*UMPS*, *HMGCR*) and (*CAD*, *HMGCR*) could improve the metabolic deviation grade by more than 22% without reducing the cell viability grade.

## Abbreviations

**symbol**
: **enzyme**
ACACA: acetyl-CoA carboxylase 1
ADSL: adenylosuccinate lyase
ADSS2: adenylosuccinate synthetase isozyme 2
ATIC: bifunctional purine biosynthesis protein ATIC
CA5A: carbonic anhydrase 5A, mitochondrial
CAD: CAD protein
CEPT1: choline/ethanolaminephosphotransferase 1
CRLS1: cardiolipin synthase (CMP-forming)
CYC_COX: cytochrome b, c complex
DHFR: dihydrofolate reductase
DHODH: dihydroorotate dehydrogenase (quinone), mitochondrial
FDFT1: squalene synthase
GSR: glutathione reductase, mitochondrial
HMGCR: 3-hydroxy-3-methylglutaryl-coenzyme A reductase
KDSR: 3-ketodihydrosphingosine reductase
NDUFS7: NADH dehydrogenase [ubiquinone] iron-sulfur protein 7, mitochondrial
PAICS: multifunctional protein ADE2
PC: pyruvate carboxylase, mitochondrial
PFAS: phosphoribosylformylglycinamidine synthase
PGK1: phosphoglycerate kinase 1
PGS1: CDP-diacylglycerol-glycerol-3-phosphate 3-phosphatidyltransferase, mitochondrial
PPAT: amidophosphoribosyltransferase
PRDX6: peroxiredoxin-6
RPIA: ribose-5-phosphate isomerase
SLC25A10: solute carrier family 25 member 10
SLC2A13: proton myo-inositol cotransporter
SLC5A3: sodium/myo-inositol cotransporter
SLC7A6: Y+L amino acid transporter 2
SPTLC3: serine palmitoyltransferase 3
TAT: tyrosine aminotransferase
TYMS: thymidylate synthase
UMPS: uridine 5'-monophosphate synthase
**symbol**
: **metabolite**
25aics[c]: (S)-2-[5-amino-1-(5-phospho-D-ribosyl) imidazole-4-carboxamido] succinate
air[c]: 5-amino-1-(5-phospho-D-ribosyl) imidazole
asp_L[m]: L-aspartate
c6crn[c]: hexanoyl carnitine
cbasp[c]: N-carbamoyl-L-aspartate
cbp[c]: carbamoyl phosphate
CE0785[m]: cis,cis-myristo-5,8-dienoyl coenzyme A
cs_d_deg5[l]: chondroitin sulfate D (GlcNac6S-Glca2S), degradation product 5
dadp[c]: deoxyadenosine diphosphate
dag_hs[c]: diglyceride
dhor_S[c]: (S)-dihydroorotate
dudp[c]: deoxyuridine-5'-diphosphate
dutp[c]: deoxyuridine-5'-triphosphate
gar[c]: N1-(5-phospho-D-ribosyl) glycinamide
imp[c]: inosine-5'-monophosphate
orot[c]: orotate
orot5p[c]: orotidine 5'-phosphate
phpyr[m]: keto-phenylpyruvate
prpp[c]: 5-phospho-alpha-D-ribose 1-diphosphate
r5p[c]: alpha-D-ribose 5-phosphate
sphgn[c]: sphinganine
trdrd[c]: reduced thioredoxin
ump[c]: uridine-5'-monophosphate

## Data Availability

The datasets supporting this article have been uploaded as part of the electronic supplementary material. The optimization code used during this study and the electronic supplementary material are available via the Dryad Digital Repository: https://doi.org/10.5061/dryad.wdbrv15s2 [52]. The data are provided in the electronic supplementary material [54].
